# Lung Function in Children with Primary Ciliary Dyskinesia

**DOI:** 10.3390/children10020290

**Published:** 2023-02-02

**Authors:** Valentina Agnese Ferraro, Raimondo Junior Castaldo, Valentina Tonazzo, Stefania Zanconato, Silvia Carraro

**Affiliations:** Pediatric Respiratory Medicine and Allergy Unit, Women’s and Children’s Health Department, University of Padova, 35128 Padova, Italy

**Keywords:** primary ciliary dyskinesia, pulmonary function test, spirometry, lung function

## Abstract

Background: Primary ciliary dyskinesia (PCD) is characterized by impaired mucociliary clearance that results in accumulation of mucus and bacteria in the airways. Lower respiratory tract infections lead to airway remodeling and lung function impairment. The aim of our narrative review is to discuss available data on lung function in PCD children, focusing on risk factors for lung function impairment. Methods: Relevant published studies searching MEDLINE/Pubmed are included in this narrative review, using these terms: “primary ciliary dyskinesia” and “pulmonary function test” or “spirometry” or “lung function”. Filters were language (English) and age of study subjects (0–18 years). Results and Conclusions: The majority of recent published studies showed normal spirometric values in PCD children, even if some authors described a pulmonary impairment. Together with spirometry, Lung Clearance Index has been applied for detecting peripheral airway disease, and it might have a role in early mild lung disease assessment. Studies on lung function trajectories after PCD diagnosis showed a significant heterogeneity, with some patients maintaining reasonably good lung function, whereas others showing a decline. Further studies are needed to analyze lung function prospectively from childhood into adulthood, and to evaluate whether lung function trajectories are affected by PCD clinical phenotype, ultrastructural ciliary defect or genetic background.

## 1. Introduction

Primary ciliary dyskinesia (PCD) is a rare genetic disorder characterized by impaired mucociliary clearance that leads to lifelong respiratory symptoms including unexpected neonatal respiratory distress in infants born at term, persistent wet cough from early infancy, bronchiectasis, chronic rhinosinusitis, and conductive hearing impairment [1,2]. Approximately 50% of patients with PCD have situs inversus and 5% of children have congenital heart disease; moreover, in patients with PCD, infertility is common, particularly in males [3,4]. A heterogeneous clinical phenotype characterizes this multiorgan disease as likely to be the result of the expression of numerous known genetic variants in PCD-related genes that are responsible for modifications in ciliary ultrastructure and function [5]. Within the airways, the abnormal ciliary beating results in excessive accumulation of mucus and bacteria in the upper and lower respiratory tract [6,7]. In the latter, recurrent infections and mucus accumulation lead to airways remodeling with the development of lung abnormalities such as bronchiectasis, peri-bronchial thickening, and mucus plugging, possibly leading to a decline in lung function [8] and to an increased work of breathing that may affect growth and quality of life [9].

The aim of our narrative review is to discuss available data on lung function in children with PCD, focusing on risk factors for lung function impairment.

## 2. Materials and Methods

MEDLINE/Pubmed database was searched for this narrative review, using the following terms: “primary ciliary dyskinesia” or “immotile cilia syndrome” or “Kartagener syndrome” AND “pulmonary function test” or “spirometry” or “lung function”. Focusing on available data on lung function in children with PCD, we considered the techniques used in the vast majority of pediatric studies: spirometry, which is a routinely used method both for diagnosis and during follow-up, and MBW. Moreover, we have focused on studies including data collected longitudinally, analyzing factors that influence lung function evolution over time. On the other hand, we did not consider other lung function tests, like impulse oscillometry or cardiopulmonary exercise tests which have been used in a very limited number of studies [10,11,12]; and we decided not to focus on other aspects of lung functioning (e.g., gas exchange and aerobic fitness). Filters applied were language (English) and age of study subjects (0–18 years).

## 3. Results

Our narrative review discusses studies published on spirometry (14 studies, see Table 1), which is the routinely used method both at diagnosis and during follow-up, and MBW (12 studies, see paragraph 3.b), which is supposed to be useful for detecting peripheral airway disease. Moreover, we discuss studies (9 studies, see Table 2) on lung function data collected longitudinally focusing on factors that may influence its evolution over time.

### 3.1. Spirometry to Study Lung Function in Children with PCD

Assessment of children with PCD traditionally includes spirometry, which is routinely used in children, from preschool age, as it is widely available and quick to perform [3]. It is useful both at the diagnosis of PCD and during follow-up, since it enables the assessment of the severity and progression of lung function impairment [2,3].

Published studies on spirometry show a substantial heterogeneity in age-matched lung function values within each age range in the PCD population [2]. Moreover, a high intra-subject variability of FEV1 (Forced Expiratory Volume in 1 s) was also described in stable PCD patients, probably due to the variable obstruction caused by secretions with cough clearance between first and following attempts [3]. In a systematic review on lung function in patients with PCD [13], authors showed that the most commonly reported spirometric indices were FEV1 and forced vital capacity (FVC), presented as both mean and standard deviations of percent-predicted values. A considerable heterogeneity for both parameters was described, probably due to genetic differences between study populations and to methodological factors related to the variability of inclusion criteria and of the methods applied to perform and evaluate spirometric measurements [13].

In 2018, using data from the international PCD (iPCD) Cohort, Halbeisen F. S. et al. [14] published the largest multicenter study on lung function in patients with PCD (991 patients) showing in the whole population a mean FEV1 and mean FVC lower than the mean reference values. When analysis was performed in the different age groups, children aged 6–9 years showed the best values compared to the other age groups (Table 1) [14]. In keeping with this, several studies showed normal mean (SD) or median (IQR) spirometric values in children with PCD, as described in Table 1 [15,16,17,18,19,20,21]. One of the studies with the highest number of PCD children enrolled (more than 100), was performed in six North American centers and found normal spirometric indices with a median FEV1 of 89%pred [15]. Another multicenter study published by Maglione et al. [16] showed that the majority of 158 children and adolescents with PCD (75%) had normal FEV1. Authors also highlighted that the first measured FEV1 and FVC were not significantly related to age at referral, but a weak, significant relationship was found with FEF25–75 (Forced expiratory flow at 25–75% of the vital capacity), probably due to a worse peripheral lung function impairment (FEF25–75) in late-diagnosed patients. Moreover, in a recent Turkish study, authors described FEF25-75 that was significantly lower in PCD compared to Cystic Fibrosis (CF), suggesting that small airways are primarily affected in PCD [22]. In keeping with this, Green et al. [23] in a cross-sectional study showed that 21.7% (13/60) and 25.0% (7/28) of patients with CF and PCD, respectively, had abnormal FEV1 and that FEV1, FEV1/FVC, and MMEF25–75 were significantly lower in children with PCD than in those with CF. Recently, Halbeisen et al. [24] in 486 children (median age at first measure: 10.94, SD 4.35) with PCD, from the iPCD Cohort, showed that lung function was reduced at baseline with a mean FEV1 z-score −1.22 ± 1.62, FVC z-score −0.74 ± 1.71 and FEV1/FVC 0.91 ± 1.44. Moreover, Rubbo et al. [25] reported a lower mean predicted FEV1 in PCD children compared with CF children up to the age of 15 years, with no difference seen in the 16- to 18-years-old category.

In conclusion, although many studies reported normal lung function there are also some recent data suggesting a reduction in spirometric values for children with PCD.

**Table 1:** Spirometric values in children with PCD. N/A = not available.

	Number of Patients with PCD, Age (Median (IQR) or Range)	FEV1	% of Patients with Abnormal FEV1	FVC	FEF25-75
Halbeisen et al., 2018 [14], mean Z-score (CI 95%)	271 children, 6–9 y	−0.84 (−1.03 to −0.65)	46%	−0.31 (−0.51 to −0.11)	N/A
	207 children, 10–13 y	−1.00 (−1.21 to −0.79)		−0.48 (−0.70 to −0.26)	N/A
	191 children, 14–17 y	−1.51 (−1.73 to −1.29)		−0.84 (−1.07 to −0.61)	N/A
Davis et al., 2015 [15], median %pred (IQR)	86 patients, 8 y (5–11)	89 (67 to 99)	N/A	N/A	68 (48 to 80)
Maglione et al., 2014 [16], mean %pred and Z-score (SD)	158 patients, 8.7 y (4.2–17.4)	82.48 (20.38) −1.37 (1.63)	35%	88.77 (20.89) −0.88 (1.68)	68.56 (29.64) −1.58 (1.53)
Boon et al., 2014 [17], median Z-score (IQR)	38 patients, 16.1 y (11.1–19.6)	−1.54 (−2.1 to −0.43)	N/A	N/A	−1.99 (−2.68 to −0.61)
Noone et al., 2004 [18], median %pred (range)	31 patients, 8 y (1–17)	85 + 3	N/A	N/A	N/A
Ring et al., 2018 [19], median Z-score (range)	22 patients, 11.8 y (6–18)	−1.28 (−2.7 to 0.1)	N/A	−0.3 (−2.3 to 0.7)	−1.9 (−2.9 to −0.3)
Constant et al., 2021 [20], mean Z-score (min; max)	6 patients, 13.8 y (11–19.2)	−1.0 (−2.8; −0.3)	N/A	−1.3 (−2.7; 0.2)	−1.5 (−2.6; 0.2)
Kinghorn et al., 2020 [21], median Z-score and %pred (IQR)	17 patients, 8.6 y (6.1–10.0)	−0.6 (−1.6; −0.2) 92% (82–98)	18%	0.05 (−0.4; 0.7) 101% (95–108)	−1.7 (−2.0; −1.0) 62 (50 to 78)
Denizoglu Kulli et al., 2020 [22], mean %pred ± SD	25 patients, 12.5 y ± 3.5	72.6 ± 15.5	66%	86.7 ± 17.4	45.8 ± 26.7
Green et al., 2016 [23], median Z-score (IQR)	28 patients, 12.4 y (10.7–14.6)	−1.3 (−1.7; −0.6)	25%	−0.4 (−1.3; 0.5)	−1.8 (−2.4; −1.8)
Halbeisen et al., 2022 [24], mean Z-score (SD)	486 individuals, 10.94 y ± 4.35	−1.22 (1.62)	N/A	−0.74 (1.71)	N/A
Rubbo et al., 2020 [25], mean Z-score (SD) and mean %pred	240 patients, 9.8 y (5.5–13.8)	−1.9 (1.4) 76.8%	N/A	−1.3 (1.5) 85.4%	N/A
Magnin et al., 2012 [26], median Z-score (IQR)	20 children, 7.6 y (4–11.7)	−1.2 (−1.8; −0.45)	N/A	N/A	N/A
Nyilas et al., 2015 [27], median Z-score (IQR) and median %pred (IQR)	30 children, 13.4 (10.4–17.1)	−0.5 (−1.6; 0.3) 99.1% (83.5%; 106.8%)	N/A	N/A	N/A

### 3.2. Lung Clearance Index (LCI) in Children with PCD

Although spirometry is routinely used in children with PCD, it is not as sensitive as LCI for detecting peripheral airway disease [17,27,28,29]. LCI is derived from a multiple breath wash-out (MBW) technique and it mirrors ventilation inhomogeneity, which might be abnormal even with normal spirometric values [2,17,30,31]. The maneuver to measure LCI is feasible in young children because it only requires tidal breathing, but the equipment is not always available and testing can be time consuming [2,20,32].

Several papers showed the role of LCI in assessing early mild lung disease in children with PCD [20,21] and a multicenter observational cohort study demonstrated the potential role of LCI in the prediction of pulmonary exacerbations and in the assessment of lung function decline [31].

In a recent systematic review, Zafar et al. [33] showed that MBW values are likely to be abnormal earlier than FEV1 in children with PCD, even if the relationship between FEV1 and LCI remains under investigation. On one side, in 27 patients with PCD (median age 11.3 years) LCI did not show a statistically significant correlation with either FEV1 or FVC [29] and in a PCD group of young adults (mean age 24.66 years) there was no relationship between LCI and FEV1 [28]. The same results have been described recently by Green et al. [24] in a cross-sectional study involving 28 patients with PCD, in which the authors showed that LCI did not significantly correlate with FEV1, while it significantly correlated with FEV1/FVC and FEF25-75. On the other side, more recently, in 42 young adult with PCD (median age 15.4 years) a moderate negative correlation was reported between the LCI and FEV1 z-scores [34], thus showing lower values of FEV1 in patients with higher values of LCI. These conclusions were confirmed in another two papers conducted using 39 patients with PCD aged 5.2–25.0 years [14], and in 47 patients with PCD aged 4.0–42.3 [27], respectively.

In addition, LCI was not able to discriminate between different lung abnormalities, as identified using chest HRCT. Data on the relationship between LCI and HRCT findings, in fact, are not univocal [2,3,34]. In some papers MBW seems promising in the early detection of lung damage [14,29,35], while other authors showed that LCI did not correlate with any HRCT parameters or MRI findings [28,36,37].

Reasons for these discrepancies between studies might be the different population analyzed, the different disease severity and the different genetic background, that probably lead to distinct PCD phenotypes. In keeping with this, a recent paper published by Irving et al. [38] described spirometry and LCI in 69 stable patients with PCD (median age 13, range 4–41) stratifying the population on the basis of ciliary ultrastructure defects and genetic mutations. They showed that cilia microtubular defects are associated with worse LCI than dynein arm defects or normal ultrastructure.

Further studies are needed to analyze PCD patients considering these differences.

### 3.3. Longitudinal Analysis of Respiratory Function in Children with PCD

Since the end of the 20th century [39,40,41], some studies have analyzed lung function data collected longitudinally in PCD patients from diagnosis and thereafter, showing that some patients maintained reasonably good lung function, whereas other have worse outcomes.

Nowadays, the scientific community is focusing on studying lung function trajectories during childhood, mainly in healthy people and asthmatic patients, through models that describe lung function at birth, lung growth during childhood, a short plateau phase and the long period of lung function decline [42,43]. As for PCD, recently, lung function trajectories were described in an international cohort of children and young adults with PCD from age 6 to 24 years [24]. Authors analyzed 4470 lung function tests from 486 individuals (20 centers) with a median of 6 lung function tests per participant and of 4.14 years of follow-up, showing an overall decline in lung function with an overall negative trend in z-scores [24]. Individual FEV1 trajectories were classified, based on the natural variability of lung function in healthy children, into three groups: those who improved over time (>0.05 z-scores per year), those who remained stable (≤0.05 and ≥−0.05 z-scores per year) and those who decreased (<0.05 z-scores per year) [44]. Lung function slopes over time showed high heterogeneity: in 39% of patients the Z-score FEV1 decreased by >0.05, in 40% remained stable and in 21% improved. A relevant variability was described between countries, mostly due to different length of follow-up time and frequency of lung function testing, and between individuals, with the steepest decline in patients with ODA (outer dynein arm) and IDA (Inner dynein arm) defects (−0.05) and the most favorable course in patients with CA (central apparatus abnormalities) [24].

In addition to this study, other authors analyzed lung function in children with PCD longitudinally, as shown in Table 2: half of them showed that lung function remained stable during the follow-up [16,40,41,45], while the other half described a significant decline of FEV1 [46,47,48] over time. These inconsistent results could be explained by the high heterogeneity between the studies as for age at first measure and duration of follow-up. One of the largest study reported that FEV1 improved over time in 10% of Danish patients, remained stable in 57% and declined in 34% [48]. Moreover, focusing on lung function growth during childhood, Davis et al. showed in 137 children with PCD in North America that lung function has a variable time course with ultrastructural defects as the main determinant of heterogeneity [46].

Finally, Fuger et al. [49] analyzed spirometric values together with blood gases and demonstrated that PaO2 and the PaO2/PaCO2 ratio similarly and significantly decreased with age both in PCD and in CF children, whereas PaCO2 increased more in CF.

**Table 2:** Longitudinal course of spirometric values in patients with PCD.

	Number of Patients with PCD and Median Length of Follow Up	FEV1	FVC	FEF25-75
Ellerman et al., 1997 [40]	12 children (median age at entrance 9.5 y) followed for a median of 7 y	Median: - at entrance to cohort 85%pred - at end of follow-up 85%pred	Median: - at entrance to cohort 72%pred - at end of follow-up 69%pred	N/A
Hellinckx et al., 1998 [41]	10 patients (mean age at entrance 15.2 y, SD 7) followed for 3–20 y	Mean change: +0.3%pred (SD 11.7)	N/A	N/A
Halbeisen et al., 2022 [24]	486 individuals (mean age at first measure 10.94 y, SD 4.35), median follow up time 4.14 y (IQR: 2.3–7.6)	Average annual change Z-score −0.06 (95% CI −0.072 to −0.057)	Average annual change Z-score −0.03 (95% CI −0.040 to −0.023)	N/A
Vallet et al., 2013 [45]	60 patients (mean age at first measure 6 y), median duration of follow-up 5 y (range 1–17 years)	No significant differences between first and last recorded evaluation	No significant differences between first and last recorded evaluation	N/A
Maglione et al., 2014 [16]	78 patients (median age at first spirometry 8.7 y, range 4.2–17.4), followed for 6 years	Mean slope: Z-score 0.05 (−0.10 to −0.01) Mean (SD) - first measurement: 82.48%pred (20.38); Z-score −1.37 (1.63) - 6 years: 83.76%pred (19.0); Z-score −1.39 (1.63)	Mean slope: Z-score −0.03 (−0.08 to 0.02) Mean (SD) - first measurement: 88.77%pred (20.89); Z-score −0.88 (1.68) - 6 years: 93.46%pred (17.17); Z-score −0.60 (1.57)	Mean slope: Z-score −0.06 (−0.11 to 0.02) Mean (SD) - first measurement: 68.56%pred (29.64); Z-score −1.58 (1.53) - 6 years: 65.22%pred (27.22); Z-score −1.81 (1.51)
Davis et al., 2019 [46]	137 children (mean age at enrollment 7.8 y, SD 4.6) followed for up to 5 y	Mean annual change: −0.57%pred	N/A	N/A
Pifferi et al., 2020 [47]	135 children (mean age at enrollment 10.24 y, SD 3.78), mean follow-up 5 y (range 1–10 y)	Mean annual change (Z-score): −0.89% per year	Mean annual change (Z-score): −0.70% per year	
Magnin et al., 2012 [26]	20 children (median age at diagnosis 7.6 y, IQR 4–11.7), median duration of follow up 15.4 y (IQR 13–20.9)	Mean annual decrease: Z-score −0.10; −0.89%pred	Mean annual decrease: Z-score −0.09; −0.50%pred	Mean annual decrease: Z-score −0.07; −1.73%pred
Marthin et al., 2010 [48]	74 children (median age at diagnosis 8.1 y, range 0–43.7), median follow-up 9.5 y (range, 1.5–30.2)	Three groups: - in 7 children FEV1%pred increased >10 percentage points - in 42 children stable - in 25 children FEV1%pred decreased >10 percentage points	Mean change from −28 to +28%	N/A

### 3.4. Factors That Impact Lung Function

Given that the progression of lung function decline is highly variable, several papers have tried to identify factors that may influence its evolution over time.

Several longitudinal studies [39,40,41] have demonstrated that lung function deteriorates before the diagnosis of PCD and then stabilizes after diagnosis and appropriate treatment, thus suggesting undertreatment as one of the possible causes of lung function deterioration and early intervention and intensive therapy as a relevant action to prevent this insidious decline [39,40,41,50]. Ellerman et al. [40] showed that lung function was significantly lower in PCD patients entering the cohort as adults when compared to the PCD patients entering as children. In keeping with this, several authors described a correlation between age at diagnosis and spirometric values, showing that late diagnosis is associated with decreased FEV1 [46,51]. In contrast, Marthin et al. [48] in a single-center longitudinal study (74 children and adults observed for median 9.5 years) showed that variation of lung function after diagnosis was not linked to either age or level of lung function at diagnosis and concluded that early diagnosis did not protect against decline in lung function. Similar results were reported by Halbeisen et al. [24] in a recent study from the iPCD Cohort, in which spirometric values were not different according to age at diagnosis of PCD.

A challenge for the years to come is the analysis of the impact of PCD chronic treatment on lung function. Recently, Kobbernagel et al. [52] demonstrated in the BESTCILIA study (multicenter, double-blind, parallel group, randomized, placebo-controlled, phase 3 trial) that azithromycin prevents lung function decline in children with PCD and reduces the need for additional antibiotic treatments, but no significant differences were shown in lung function between children treated with azithromycin and those with placebo, probably due to the short azithromycin treatment period (6 months) analyzed.

Respiratory infections are among the major determinants of morbidity and mortality in patients with PCD and they are correlated to functional decline. During exacerbations, mostly due to Haemophilus influentiae and Streptococcus pneumoniae in children [53], PCD patients present a significant reduction in FEV1 [54] and approximately 25% of children with PCD fail to recover to baseline lung function after treatment [55]. A single center, retrospective, cross-sectional study, performed in both adults and children with PCD, showed that PCD patients with more than two exacerbations per year have a more severe disease in terms of structural damage and lung function impairment [56]. Pseudomonas aeruginosa (PA) is an important pathogen in PCD, from which the colonization rate increases with age [53,56]. Few studies have investigated lung function in children with PA colonization, showing that FEV1 did not differ between colonized and non-colonized patients [45,56,57]. Moreover, in a retrospective study, Cohen-Cymberknoh et al. [58] showed that the magnitude of decline in pulmonary function was similar in colonized and noncolonized PCD patients, although lung PA colonization is associated with more severe disease as shown by the FEV1 and CT score.

Long-term changes in spirometric values have been related to the underlying ultrastructural defect or genotype [51]. Davis et al. [46] showed that PCD children with IDA/CA/MTD (inner dynein arm/central apparatus abnormalities/microtubular disorganization) defects had poorer lung function at the time of enrollment and at all ages compared with those with isolated ODA (outer dynein arm) defects, despite a younger age at diagnosis. In keeping with this, recently, Halbeisen et al. [24], in 486 children from the iPCD Cohort, showed that spirometric values were associated with ultrastructural defects: patients with MTD had the worst baseline FEV1 z-scores (−1.75), followed by patients with CA (−1.38) and ODA defects (−1.27), while patients with combined ODA and IDA defects (−0.60) had the best values at baseline but the steepest FEV1 decline overtime. The same results were confirmed by Pifferi et al. [47], in a longitudinal cohort of 135, described patients with IDA/CA/MTD defects or CCDC39 and CCDC40 biallelic mutations that had a worse prognosis, while lung function in biallelic DNAH11 mutations was less impaired. As for static lung volumes, Pifferi et al. [59] showed that children with IDA/CA/MTD defects or CCDC39 and CCDC40 mutations had the greatest increase in hyperinflation, whereas those with ODA defects and normal ciliary structure or DNAH11 and DNAH5 mutations had less severe changes. These findings are remarkable, mainly because they may be considered to establish when children with PCD must be treated more aggressively.

Moreover, a retrospective study showed that PCD patients with a loss-of-function mutation in the Mannose-binding lectin (MBL), a first line host defense protein of innate immunity, have a greater decline in FEV1 than patients with MBL sufficiency [58].

Another factor that could influence lung function evolution is airway remodeling induced by the impaired balance between matrix metalloproteinases (MMPs) and their tissue inhibitors (TIMPs). In particular, a cross-sectional evaluation showed that the sputum level of MMPs was associated with air flow limitation and structural changes in chest computed tomography, and lower concentrations of TIMPs were associated with progressive loss of lung function [60]. In addition, changes in the thickness of the reticular basement membrane (RBM) (a layer of extracellular matrix below the airway epithelium composed of a thin basal lamina and thicker reticular lamina) and lung function has been recently studied in patients with PCD, CF and bronchial asthma [61]. Authors showed that RBM thickening occurs both in eosinophilic (bronchial asthma) and neutrophilic (CF and PCD) airway inflammatory disorders and it is related to ventilation inhomogeneity (studied using nitrogen MBW) in CF and PCD [61].

Finally, nutritional status probably influences lung function. Several studies showed that PCD children with lower body mass index (BMI) z-scores have lower FEV1 z-scores [9,16,25], suggesting that poor nutrition is associated with impaired lung function in these patients, leading to the hypothesis that interventions on nutritional status may have a positive impact on lung function in children with PCD. Similar results are well-described in children with CF [62], in whom, indeed, the poor nutritional status is mainly associated with pancreatic insufficiency. The available data on PCD patients suggest that suppurative lung diseases may impact on nutritional status and/or vice versa, with an effect not mediated by pancreatic dysfunction.

## 4. Conclusions

Despite a high heterogeneity, most of the published studies have shown normal spirometric values in children with PCD. Nonetheless, some recent papers suggest that in PCD children spirometric values might be more impaired than expected; even if the clinical relevance of the reported cases are statistically significant, results remain to be assessed. Further studies are needed to evaluate whether statistically significant changes in lung function influence, or not, clinical symptoms. Besides spirometry, LCI has been applied in children with PCD for detecting peripheral airway disease. LCI has a promising role in the assessment of early mild lung disease, even if its relationship with spirometric parameters (FEV1, in particular) and HRCT scores is not clear.

Some studies that have analyzed lung function trajectories after the diagnosis of PCD showed a significant heterogeneity, with some patients maintaining reasonably good lung function whereas others showing a decline.

Undertreatment is one of the possible causes of lung function deterioration, thus suggesting that the insidious decline in respiratory function in patients with PCD may be prevented by early intervention and intensive therapy. Moreover, the long-term trends in spirometric values has been related to the underlying ultrastructural defect or genotype, with some mutations such as CCDC39 and CCDC40 being associated with a worse prognosis and others such as DNAH9, DNAH5 and DNAH11 with milder disease.

A limit of our narrative review is that we did not analyze other aspects of lung function (e.g., gas exchange and aerobic fitness) or other methods for lung function testing (e.g., impulse oscillometry and cardiopulmonary exercise testing), focusing instead on spirometry and multiple breath wash-out, which are the techniques used in the vast majority of studies investigating lung function in PCD children.

Further studies are needed to analyze lung function prospectively from childhood into adulthood, and to evaluate whether lung function trajectories are affected by PCD clinical phenotype, ultrastructural ciliary defects or genetic background.

## Data Availability

Not applicable.
